# Exploring older migrants’ meaning-making of ‘happiness’: “*The main thing is health. Young people might say otherwise.”*

**DOI:** 10.1080/17482631.2023.2300873

**Published:** 2024-01-07

**Authors:** Phlix Micheline, Jan Vanrie, Ann Petermans, An-Sofie Smetcoren

**Affiliations:** a Architecture and Arts, UHasselt – Hasselt University, Diepenbeek, Belgium; b Vrije Universiteit Brussel (VUB), Faculty of Psychology and Educational Sciences, Brussels, Belgium

**Keywords:** Meaning-making, well-being, happiness, older migrants, health, independence, social relations

## Abstract

**Purpose:**

As our ageing population is growing and diversifying, it is important to gain insight into the well-being of older migrants. However, the meanings of happiness can vary cross-culturally. Therefore, prior to exploring older migrants’ happiness, their meaning-making of “happiness” should be explored. This way, cultural or individual variations can be considered when analysing older migrants’ happiness. Not only migration background but also age could influence the meaning of well-being. For example, the meaning of well-being can change as people grow older. Therefore, both migration background and age are considered in exploring older migrants’ meaning-making of happiness.

**Methods:**

To do so, in-depth interviews with older migrants (*n* = 22) from various ethnicities were conducted in which their meaning-making of happiness was questioned via a semi-structured interview guide.

**Results:**

After analysing the results via thematic analysis, three overarching themes are discussed: (1) happiness associations, (2) happiness-pursuing strategies, and (3) happiness obstructions. The analysis then further focuses on the role of migration background and ageing on the meaning-making of happiness.

**Conclusions:**

Participants’ meaning-making of happiness seems strongly imbued with age-related references. On the contrary, the impact of migration background is rather limited. To explain this difference, the value of incorporating participants’ life course experiences emerged.

## 1. Introduction

The older population is growing and diversifying (Ciobanu et al., 2017), including in Belgium. Due to labour migration, former Belgian mining municipalities have a high share of older adults with a migration background (i.e., 1st and 2nd generation migrants aged 60 and older). Therefore, it is important to gain insight into the housing needs and well-being of older adults with a migration background (or: older migrants) (Phlix et al., 2023a). To explore such questions, researchers should be mindful of cultural variances in older migrants’ meaning-making of happiness as its meaning is culture-dependent and can vary across cultures, individuals, and nations (Diener et al., 2018; Oishi & Gilbert, 2016; Shin et al., 2018). Research shows that migrants use their culture of origin as a reference to reflect on what healthy and successful ageing means to them (Chowdhury et al., 2023; Torres, 2007). This shows the possible influence of migration background on the meaning-making of “happiness”. In light of such findings, Manasatchakun et al. (2016) argue that adopting a culturally sensitive perspective is important to fully understand older adults’ meaning-making of, for example, healthy ageing. Thus, it points to the need to explore migrants’ meaning-making of “happiness” to understand their perspective in happiness research. This is especially important as one’s meaning-making of happiness influences their actual happiness levels (Shin et al., 2018). Therefore, this paper aims to explore older migrants’ meaning-making of happiness. Meaning-making concerns the individual process of understanding the world (Alea & Bluck, 2013; Kurzman, 2008). This process is dynamic and ongoing as meanings can change, for example, through reinterpretation of situations over time or through interaction with others (Boehmer et al., 2007; Manning & Kunkel, 2014). The latter highlights the importance of reference points to construct meaning (Noguchi, 2020).

Some researchers use the terms “well-being” and “happiness” to refer to the same conceptualization. However, there are also many conceptual nuances and differences within the field (see e.g., Diener et al., 2018). Therefore, when referring to previously published work, we adhere to the term used by the original authors of the referenced work. Happiness is often associated with health and social relationships (Bojanowska & Zalewska, 2016). Research by Lyubomirsky et al. (2005, 2007) has identified three general determinants of happiness: the genetic set point, life circumstances (e.g., gender, age, ethnicity, religion), and intentional activities (e.g., attitudes and behaviours to deal with life circumstances)—of which the weight is under debate (Brown & Rohrer, 2020). Concerning the intentional activities contributing to happiness, it is crucial that these activities fit with the individual (i.e., person-activity fit), and an effort is required to initiate and maintain the activity (Lyubomirsky et al., 2005). Although positive life changes tend to have a temporary positive impact on people’s well-being (Diener et al., 2018), Sheldon and Lyubomirsky (2012) found that continued appreciation of the positive life change can contribute to a more sustainable positive impact on happiness. This shows that people can—to a certain extent (Brown & Rohrer, 2020)—increase their happiness. Culture also plays a role in this matter (Sheldon & Lyubomirsky, 2019).

### 1.1. Happiness & ageing

People’s conceptualization of happiness can change over time (Oishi & Gilbert, 2016) and with age (Bojanowska & Zalewska, 2016; Laaksonen, 2018). Older adults weigh and conceive happiness-influencing factors differently than their younger counterparts. For example, older adults view the future differently, as less open and consider their life mostly accomplished. Hence, the past becomes more important to their well-being and can be used to balance out present negative experiences (e.g., mobility decline) (Klausen, 2020). Also, older adults draw from past sources of meaning, such as work, social relations and family for meaning-making in later life (Halama et al., 2021). In addition, independence grows in importance to older adults’ well-being and norms and values may change (Klausen, 2020).

The literature on ageing and well-being points to a U-shaped pattern, in which well-being declines in middle age and rises again as people grow older (Blanchflower & Oswald, 2008). However, this U-shaped happiness pattern has been challenged and remains a question (Toshkov, 2022). Even if older adults experience higher levels of well-being than younger adults, they face sharper declines in well-being, especially in very old age (Jivraj et al., 2014). Another study found that the U-shape holds up in young and middle age, but instead of an ongoing increase, people’s happiness stagnates after 50 and even declines in very old age (Becker & Trautmann, 2022). Studies point out the individuality and complexity of various intersecting characteristics in researching happiness. Recent research shows that income has a moderating influence on the relationship between age and happiness. The U-shaped pattern was found among people from the middle class. However, for people with a low income, a “hockey stick” shape was found instead of a U-shaped pattern, i.e., a substantial decline in age 50–55 with only a small increase in older age (Toshkov, 2022). Gender also affects the U-shape, as it holds up better for men than women. The increase in happiness stops at around 70 for men and around 80 for women (Laaksonen, 2018).

The literature also points to a strong relationship between age, health and subjective well-being, as health affects well-being (Steptoe et al., 2015). For example, a decline in health—often associated with ageing—can account for a decline in well-being in later life (Jivraj et al., 2014). Interestingly, Steptoe et al. (2015) found that despite an age-related decline in health, older adults experience higher levels of well-being than their younger counterparts. This could be explained by older adults’ greater knowledge of what makes them truly happy (i.e., “educated desires”) (Diener et al., 2018), leading them to pursue personally relevant goals (Lyubomirsky et al., 2005). Such high levels of well-being in later life could, in turn, be a protective component for older adults’ health. However, the relationship between age and happiness is affected by many more aspects than health, such as income (Toshkov, 2022), gender (Laaksonen, 2018), and social relations. The latter changes as people age (Steptoe et al., 2015). For example, social exclusion in later life negatively impacts older adults’ well-being. However, again, this relation is moderated by other variables. For example, being male, having low income, reporting poor health or being over 80 increases the chance of social exclusion (Prattley et al., 2019). As the ageing process is embedded within a social context, changes within this context (e.g., social exclusion, loss of social support) can affect the life satisfaction of older adults (Kodzi et al., 2011).

### 1.2. Happiness & migration

Migrant status has been identified as a positive predictor for well-being (Ryff et al., 2003), also compared to migrants’ counterparts who remained in the country of origin. However, this contrast can be explained by a difference in happiness between these groups prior to migration (Bartram, 2012). Hence, migration is not necessarily a predictor for higher levels of happiness. Other research on older migrants points explicitly to the possible negative influence of migration background on their well-being through accumulated disadvantages (Buffel, 2017). Moreover, the effect of migration on happiness significantly varies by country (Bartram, 2012). Overall, migrants with high levels of happiness tend to remain in the country of settlement (Shamsuddin & Katsaiti, 2020). Looking at older migrants specifically, “age” and “migration” intersect as possible influences on well-being. For example, visiting the country of origin and engaging with the traditional culture positively relates to older migrants’ life satisfaction (Chappell, 2005). In addition, research shows that migrants have a positive approach towards ageing, positively impacting their well-being in later life (Moriarty & Butt, 2004). However, a recent study suggests that perceptions of ageing also vary cross-culturally (Cramm & Nieboer, 2018). Moreover, even within a culture, there are variations in happiness-related influences (Oishi & Gilbert, 2016). Culture is considered an important factor in people’s well-being. For example, people appraise situations differently, partly because their culture and influences on well-being are dependent on their culture (Diener et al., 2018). Not only do the predictors of well-being vary across cultures, but the conceptions and meaning of happiness are also culture-dependent (Oishi & Gilbert, 2016; Uchida et al., 2004). However, happiness has some universal basis (e.g., satisfaction of basic needs). Fulfilling one’s social needs seems to be a universal, cross-cultural condition for happiness (Shin et al., 2018). Research on older migrants highlights the possible obstructive role of the language barrier in creating social relations (Phlix et al., 2023b). However, the meaning-making of happiness is influenced by culture (Diener et al., 2013; Pflug, 2009). For example, happiness is defined differently in individualistic and collectivistic cultures (Pflug, 2009; Uchida et al., 2004), and the culture in which people are socialized also plays an important role in nurturing well-being. Moreover, the strength of influences on happiness is culturally dependent (Diener et al., 2013), as well as the effectiveness of happiness-increasing strategies. For example, Layous et al. (2013) show that some strategies (e.g., gratitude expression) are more effective in some cultures than others. Given the many variations in the meaning and (strength of) predictors of well-being, it is important to consider cultural differences in research on well-being.

Religion, intertwined with culture, has been considered a predictor for higher well-being. For example, social support derived from religious communities positively affects well-being. Also, religion forms a lens through which a successful lifestyle is defined (Kodzi et al., 2011), affecting the meaning-making of well-being. For example, Islamic and Western conceptions of happiness can differ. Whereas autonomy is highly valued in Western culture, it is not among Muslims. This difference can be explained by the importance of the Islamic religion in Muslims’ meaning-making of happiness (Joshanloo, 2013). However, the impact of religion is not universal, as its effects are culturally and context-dependent (Diener et al., 2018). For example, in wealthy countries, religious people do not have higher levels of well-being than non-religious people (Diener et al., 2011). Moreover, the authors also point to the religion-environment fit, i.e., religious people in a religious environment fit in, whereas non-religious people in a religious environment do not, affecting their well-being. Therefore, we argue that, in light of migration, this religion-environment fit—or lack thereof—could play an essential role in migrants’ well-being.

### 1.3. The present study

The literature highlights the possible influencing role of migration background on migrants’ meaning-making of well-being (e.g., Cramm & Nieboer, 2018). This can considerably impact the interpretation of data in happiness research concerning migrants. For example, migrants might adhere to different meanings of happiness than non-migrants in the country of settlement and, therefore report high levels of happiness. It is crucial to take such variations into account to avoid distorted conclusions. However, such critical notions have not received sufficient attention (Joshanloo, 2013). Moreover, while culture plays a significant role in shaping the meaning-making of well-being, it is not the paramount factor influencing it (Hajdu & Hajdu, 2016). The meaning-making of happiness is also impacted by age (Diener et al., 2013; Laaksonen, 2018). For example, in their cross-cultural study with older adults from America and Congo, Westerhof et al. (2000) show that similarities (e.g., more collectivistic meaning-making in later life) and differences (e.g., different cultural understandings of “old age”) in meaning-making are not only affected by culture but also by age. Moreover, given the often lengthy period older migrants have spent in the country of settlement, one might argue that their meaning-making of happiness is also influenced by the culture of the receiving country, adding to the complexity of older migrants’ happiness. For example, Chowdhury et al. (2023) show that older migrants’ perspective on the ageing process is influenced by their dual belonging to multiple cultures. Therefore, we argue that it is crucial to map older migrants’ individual meaning-making of happiness to comprehend their happiness. Furthermore, reviewing the literature, the complexity of influences on people’s happiness becomes clear. For example, many moderating factors affect the relationship between age and happiness. Therefore, we argue that it is imperative to acknowledge the intersecting influences in research on older migrants’ meaning-making of happiness, in which a bottom-up, open research approach (e.g., free association) is deemed valuable (Shin et al., 2018).

Thus, given the age-related and cultural variations in the meaning-making of happiness or well-being (Bojanowska & Zalewska, 2016; Diener et al., 2018; Klausen, 2020; Oishi & Gilbert, 2016; Shin et al., 2018) and the complexity of intersecting influences (e.g., gender, age, migration background, income) as highlighted above, this paper aims to explore older migrants’ meaning-making of happiness in an open, associative manner.

## 2. Materials and methods

The overarching goal of the research project was to gain insight into older migrants’ subjective well-being.^1^ Considering the individual and cultural interpretations of well-being (Diener et al., 2018; Oishi & Gilbert, 2016), we conducted interviews, of which the first part included some questions to gain insight into older migrants’ meaning-making of well-being—which is this paper’s focus.

To explore older migrants’ meaning-making of subjective well-being, 19 in-depth semi-structured interviews with 22 older migrants (due to 3 couple interviews) were conducted. Participants were recruited via community and municipality workers, established relations from previous studies, and snowballing). The search criteria were; (1) 60 years and older, (2) migration background (1st, 1.5,^2^ or 2nd migration generation), living in the province of Limburg (Belgium), and living in their long-time home.^3^ This resulted in a diverse sample of 22 older migrants (see Table I) from various ethnicities (i.e., Turkish, Russian, Dutch, Dutch-Indonesian, Italian, Polish, Moroccan, Spanish and Greek). The interviews were conducted in Genk and Maasmechelen, former mining municipalities with a rich migration history, in Dutch. All participants signed informed consent and could choose a pseudonym themselves. In doing so, some indicated the preference to use their real first name, in which the researchers followed their wishes. In addition to obtaining informed consent, the data was handled confidentially in line with the ethical advice and approval from the Ethics Committee of Hasselt University.

**Table I.** Overview of participants.

Participant	Age	Gender	Migration background	Migration generation
Nico	69	Male	Italian	1.5
Renata	69	Female	Italian	1^st^
Marouan	65	Male	Moroccan	1^st^
Aicha	Around 60^4^	Female	Moroccan	1^st^
Vera	62	Female	Russian	1^st^
Fatma	62	Female	Turkish	1^st^
Elena	71	Female	Italian	1.5
Rietje	75	Female	Dutch	1^st^
Giacomo	75	Male	Italian	1.5
Krysia	85	Female	Polish	2^nd^
Jan	75	Male	Polish	1.5
Lotka	72	Female	Polish	2^nd^
Massimo	64	Male	Italian	1^st^
Isabella	59^5^	Female	Italian	2^nd^
Halina	71	Female	Polish	1^st^
Liliana	76	Female	Polish	2^nd^
Paola	65	Female	Italian	2^nd^
Inge	63	Female	Dutch-Indonesian	1^st^-2^nd^^6^
Mauro	65	Male	Italian	1.5
Piero	66	Male	Italian	1.5
Antonia	66	Female	Spanish	1.5
Theo	70	Male	Greek	1.5

As opposed to “happiness”, the term “well-being” is rather scientific (Lyubomirsky, 2007), and the meaning of “happiness” and “well-being” can be inconsistent across languages as is the case for Dutch and English (Veenhoven, 2013), which can affect the measurement (Veenhoven, 2012). In Dutch, the term “happiness” is preferred by researchers as “well-being” is often associated with welfare work (Peeters Weem, 2011). So, to ensure clear communication, the term “happiness” was used to operationalize “well-being” (in Dutch) in the interview guide. Various open questions were posed, all probing older migrants’ meaning-making in slightly different ways: (1) What do you think of when you hear the word “happiness”? (2) What do you need to be happy? (3) What does “being happy” mean to you? (4) How would you define “happiness” for yourself? Especially the first question allows for free association. Such an open research approach grants access to participants’ associative network and spontaneous thoughts on happiness. In doing so, this research design steers away from the often restricted expression of participants’ meaning-making in happiness studies (Shin et al., 2018).

After introducing the interviewer (i.e., the first author) and the research project and signing the informed consent, some introductory questions regarding demographics (e.g., age and migration background) were asked. Next, the four aforementioned interview questions to inquire about older migrants’ meaning-making of well-being were posed. After the interview, participants were asked if there was anything they would like to add to the conversation. The interviews were then audio-recorded and transcribed in Word. The interviews were analysed in MAXQDA software via the steps of thematic analysis (Braun & Clarke, 2006) as this inductive analysis method can be used to identify “patterns within personal or social meaning around a topic” (Clarke & Braun, 2017, p. 1). After transcribing and reading through the transcripts, all data relevant to the research question were coded in an open manner. Second, numerous codes from the first phase were organized (e.g., deleting redundant codes and creating themes with subcategories). Third, the existing coding tree was tested and revised where necessary. For example, initial codes on the meaning-making of happiness such as “being together with family” were categorized as “social relations”. This process resulted in three overarching themes reflecting the participants’ main discussions on their meaning-making of “happiness”: 1) happiness associations (including the code “social relations”), 2) happiness-pursuing strategies, and 3) happiness obstructions. Finally, in light of the focus on older migrants, the role of “age” and “migration” in participants’ meaning-making of happiness was analysed.

## 3. Results

Below, the results will be discussed through the three identified overarching themes: (1) happiness associations, (2) happiness-pursuing strategies, and (3) happiness obstructions. Quotes are used to discuss the results to adequately reflect older migrants’ meaning-making.

### 3.1. Happiness associations

Many topics emerged when participants were asked about their meaning-making of “happiness”. However, the analysis revealed some prevalent topics in almost all interviews: health, family, social relations and independence.

#### 3.1.1. Health

Being healthy was a prominent theme that emerged from the interviews. It was often the first thing participants mentioned in discussing the meaning of happiness, and it was repeatedly emphasized throughout the conversations. Some participants reflected on the increasing importance of health as they age. Although most participants were in relatively good health, some had encountered or currently faced health adversities. This, in combination with the nearing of a period in life in which health and mobility are more likely to decline, participants seemed to be more aware of the importance of being healthy and being grateful for their health. In other words, participants used such health-related experiences and the growing importance of their health as a reference point in their meaning-making of happiness.

“I am going to say it again. The main thing is health. Maybe because we are older. Young people might say otherwise.”

      –Jan (75), Polish migration background

Participants also indicated that health is a factor beyond their control. For example, Aicha drew a parallel between poor health and financial concerns. In the case of the latter, there are safety nets (e.g., social security in Belgium) which she considers not present in poor health. In line with this, Halina said: “*We do not have to win the lottery to be happy. Because if you get sick, then those millions are of no use. Even Jackie Kennedy did not have enough money to beat cancer*”.

Finally, health seems closely related to independence—another prominent association in participants’ meaning-making of happiness—which is further discussed. Participants’ accounts indicate that being in good health grants access to independence. Being in good health enables participants to participate in activities they like and, more importantly, makes them happy. For example, Paola loves to go on bike rides and uses her bike to visit her children. In addition, Piero mentions that being in good health ensures mobility. In his view, having to depend on someone else to get around (e.g., “*being in a wheelchair*” – Piero) would negatively affect his happiness. Nico also associates poor health with an increased dependence on others, a circumstance he believes negatively affects his happiness. Finally, Liliana reflects on her past inability to take part in activities due to an illness, which harmed her happiness:

“Staying healthy and independent for as long as possible. That is what matters to me […] I am telling you, I don’t want to go through that [being sick] again. I was a wreck. I couldn’t do anything. I didn’t even think of that [being happy]. I was just sick. But whether I was happy? No. You can’t be happy if you can’t do anything.”

     –Liliana (76), Polish migration background

#### 3.1.2. Family & social relations

INTERVIEWER: “What do you think of when you hear the word “happiness”?

ELENA: “I think of the word “family”. That I can sit here with my family, with my husband, my children, grandchildren […] First and foremost, good health and family. That is also important to me, good health for the whole family.”

     –Elena (71), Italian migration background

Participants emphasized the importance of social interactions for their happiness, particularly with significant others such as a spouse, (grand)children, family, friends and neighbours. The intensity of these interactions varied among individuals, with some valuing deep familial connections while others emphasized the significance of superficial interactions, such as greetings with neighbours. Aicha, for instance, considered social interactions essential to her happiness, especially since her family resides in Morocco: “*For me, that is mandatory. The doctor told me that if I do not have contact with others, I become sick, depressed* […] *We are alone in the apartment, no children, we do not have much family here*”. Although the family is most relied on for in-depth social relations, neighbours are key actors in providing volatile interactions (e.g., small talk, greeting each other) and social support. For example, Elena derives not only a sense of company but also a sense of security from the presence of her neighbours, as she knows she can rely on them in an emergency. Interestingly, Fatma referred to her Turkish culture in her meaning-making process of happiness. She points to a Turkish saying in discussing the importance of having interactions with neighbours:

“We see each other and greet each other, but if they don’t even say hello, I would think: “Where have I ended up?” We have this saying [in the Turkish culture]: “Before you buy your house, you buy neighbours”. […] What do I need [to be happy]? You know what it is, people around you who also support you a little bit. When they say “good morning”, that gives you happiness.”

    –Fatma (62), Turkish migration background

Participants consistently expressed concern for others as a crucial aspect of their own happiness. This value of caring for others frequently emerged among the participants, indicating its significance as a core principle for many of them. For Marouan, this related to his religion:

“Everything I want for myself, I have to do that for others too. That is also in the Quran; everything you do for yourself, you should do for others. If I see a poor person, I go and help them. You have to do that. You should not be selfish and think only of yourself. If you are happy, try to make other people happy too.”

  –Marouan (65), Moroccan migration background

Besides religion, age also seemed to influence participants’ concern for others. For example, Fatma refers to a change in her view as she ages. Now that she is older, she is more concerned with her children’s happiness. Many other participants also considered the happiness and health of their (grand)children as a crucial condition for their own happiness. For example, Elena said: “*Their happiness is our happiness. If I know they are not well, then I am also unhappy*”, and Antonia very similarly said: “*If my children are happy, then I am happy. From the moment one of your children is struggling, that creeps under your skin*”.

In talking about the meaning of happiness, participants often referred to other people’s experiences to shape or position their views. Second-generation migrants referred to the challenges their parents or other first-generation migrants had to overcome. For example, Fatma referred to the migration experience of a first-generation Turkish neighbour, which was very different from hers. Fatma also referred to her parents’ struggles with Dutch. Elena shared how she saw her parents suffering after migrating to Belgium. Theo also used his parents’ migration experience to position his view on happiness in relation to being a migrant, pointing to the role of migration generation in migrants’ happiness:

“I think my parents’ happiness was not as great as mine because they came to Belgium at too old an age. I came here as a little plant and grew up on Belgian soil. But my parents originate from a different soil, and they have been transplanted to a less fertile soil. They were able to flourish, but comparatively, I think, less than me because they grew up in a different culture, and they wanted to overturn that culture, but they couldn’t. That culture was ingrained in them, and it wasn’t ingrained in me.”

      –Theo (70), Greek migration background

#### 3.1.3. Independence

In asking participants about the meaning of happiness, many referred to their ability to be independent. Participants described this in various ways, for example, not being a burden to anyone, being free, not having to listen to anyone, being mobile, and doing whatever they want. For Theo, being independent is an indicator of happiness. Upon asking him about his view on losing some of his independence, he indicated that he would be unhappy in such a situation. He further elaborated on the importance of being independent: “*I just want to decide for myself how I live. So there are two ways of living: Being lived or living. We decide for ourselves what we like to do. If we want to go to the coast tomorrow, we will go there*”. Being independent also allowed participants to participate in activities such as work and hobbies that fit their personal preferences. Many participants indicated that their work had given them a strong sense of fulfilment. Participants linked their work to Belgium, their country of settlement. For example, in discussing his relationship with Poland, Jan referred to his work and pension in Belgium as a stable foundation for his happiness. For some participants, their job was a means to obtain goals that made them happy:

“I worked eight hours to get by and those other four hours to earn some extra savings, to be happy.”

     –Theo (70), Greek migration background

In addition to working, participating in hobbies and activities that fit one’s personal interests was considered valuable to happiness, according to many participants. For example, Antonia said she loves to knit, Renata mentioned going on trips with her friends and volunteering at the community centre, Halina highlighted her love for walking and dancing, and Jan enjoyed playing music and is part of a band. Some Muslim participants referred to the importance of being able to practice their religion and used their beliefs to give meaning to their happiness:

“For me, being happy is for a definite time because we will not be on this earth forever. Nobody knows when you are going to… only God knows. We came here for a purpose, and we have to exercise the purpose. I am a Muslim, I go to the mosque […] I am happy when I am praying together with my family.”

   –Marouan (65), Moroccan migration background

Moreover, being able to participate in personally meaningful activities seems to relate to a sense of control, which is crucial to many participants’ happiness. For example, Piero said that being able to take part in his preferred hobbies contributes to his happiness. Also, in discussing her happiness, Krysia talked about the joy of knitting as it is a creative outlet. However, in further discussing this, the impact of ageing on her agency in managing her activities seeps through. She regretted losing the ability to be very active, and it seems she feels no control over this matter and, therefore accepts the situation for what it is. This points to the impact of ageing on older migrants’ mindset and ability to participate in meaningful activities.

“I have always been very creative. A lot of that has fallen away. My day goes by so quickly that I am sometimes surprised that it is already done. I used to be able to do a lot. Sometimes, my husband said I was like a Duracell. I could do many things simultaneously, which I can’t do now. I don’t find that… It is what it is […] I used always to be dressed to leave. That has fallen away since I stopped interpreting. I still get dressed, but I don’t have to anymore. It has changed. Without wanting to, there is no more “having to”.”

    –Krysia (85), Polish migration background

### 3.2. Happiness-pursuing strategies

In participants’ meaning-making of happiness, they mentioned various strategies they employ to achieve happiness. The first strategy that was discussed concerned making an effort. The interviews pointed out that many participants refer to putting in an effort—either active or passive—to be happy. First, one key strategy was adopting a mindset that individuals can and should actively work on happiness. Theo exemplified this by actively countering the influence of his migration background. As a teenager, he consciously chose Belgian culture over Greek culture, as he considered the latter to obstruct his happiness.

“Being a migrant does not determine who or what you are. Who do I want to be? Do I want to be Ali, Mario, or Theo, who lives in Belgium? Or do I want to be Ali the Turk, Mario the Italian, or Theo the Greek? […] If I use my Greek style to brand myself within that community, I am Theo the Greek. Then I am no longer Theo, who lives in Belgium. That is a choice you make yourself. That stamp will not be put on you. You put that stamp on yourself.”

      –Theo (70), Greek migration background

The second strategy discussed concerned engaging in activities that personally make you happy. For example, Jan writes postcards to friends all around the world. He shares how it makes him happy to put the cards he receives back on his wall. Paola gives another example:

I make the best of it. The first thing I do is turn on the radio. Beautiful music that makes my day. You have to break the silence. You have to do it yourself. I also have days when I am sad and things are not going as well because I have had so many setbacks in my life. But you have to get over that. Then I stay at home with a book, or I watch TV. Then I try to put on TV channels that make me happy.

     –Paola (65), Italian migration background

The third strategy concerned being satisfied. More specifically, being satisfied with one’s current situation, being grateful for what you have, and not always wanting more were mentioned multiple times throughout the interviews as a more passive strategy to pursue happiness. Many participants were happy with their current situation. For example, Marouan indicated he did not want luxurious things as he was happy with his family. Also, Jan said he needs a car to get places, but this does not need to be an expensive car. Other participants considered happiness to be a result of inner satisfaction. This entailed being aware and grateful for what you have and not being jealous of others. Fatma connected this notion to health:

“We have to be happy with what we have. You can thank God that we are still healthy. If you go to the hospital for intensive care, you realise your life’s value. Or imagine you are blind. We can see now, but we are bored. How are you bored? Look around. Look at those trees. Each one is a different colour. So colourful. How nice that we can see!”

    –Fatma (62), Turkish migration background

However, being satisfied with what you have also entails accepting that certain things will not be possible anymore. For example, some participants referred to financial restrictions due to their limited pension. To cope with this, being satisfied was employed as a strategy to counteract a negative impact on their happiness. For example, Rietje loves travelling but can no longer go on international trips due to her limited pension. Therefore, she now takes smaller trips in Belgium which she is also satisfied with. Closely related to this is accepting the ups and downs of life. Some participants elaborated on setbacks in life. It seems that if participants came to terms with these setbacks, it did not negatively affect their happiness (anymore).

“Ups and downs […] since you [his wife] had that stroke, life has changed. Not that it has made me unhappy. She is still my wife, but we have to adapt. Our household, make some arrangements, she has to do this, and I have to do a bit more of that. There has been a change in that. When she was healthy, we could jump together. Now I jump alone, so to speak.”

      –Jan (75), Polish migration background

### 3.3. Happiness obstructions

As participants discussed the meaning of “happiness”, they also drew on negative experiences or instances in which they did not feel happy. The analysis revealed that such obstructions to happiness were related to various reasons, which were either migration-related, agency-related or socially related. In what follows, we elaborate on each of these.

First, some participants explicitly referred to migration-related obstructions to their happiness. Some participants referred to the first years of migration as an unhappy period in their lives. For example, Inge said: “*In the beginning, I thought this was all strange. I didn’t like that. This was a whole different neighbourhood, very different from our home*”. Participants recalled their own or their parents’ past experiences with a language barrier. This created difficulties in creating and maintaining social relations but also created a degree of dependency on others, which are—according to participants—both important elements to their happiness, as discussed above. One participant indicated that she felt homesick in her country of origin. Although Renata would like to move back, her husband does not want this, which leaves her torn. In an ideal world, she could live in both Belgium and Italy. Also, Theo was aware of his migrant status, something he considered to be a negative influence on his happiness, which he actively countered. Interestingly, most of these migration-related happiness obstructions lie in the past. Over the years, most participants started feeling good in their “new” surroundings. Parallel, the obstructive influence of their migration background on their happiness was rather limited in participants’ present experiences.

Second, agency-related obstructions to happiness concern insecurity, the absence of amenities nearby (e.g., ATM) and the inability to be independent. In discussing the meaning of happiness, some participants reflected on (the lack of) independence in relation to their happiness. Paola considers her independence an integral part of being happy: “*If you are not independent anymore, you are no longer human* […] *I would be very unhappy*”. Furthermore, some participants consider insecurity to be a happiness obstruction. For example, Giacomo refers to the need to have enough money to live a comfortable life: “*Otherwise, your happiness will suffer*”. Vera refers to the insecure period in her life right after migrating to Belgium, as she was unsure whether she could stay in Belgium and what her future would hold. She also stated that she was unhappy during this time in her life due to this insecurity. Interestingly, Marouan also referred to his first years in Belgium as an unhappy time because he felt “trapped” as there were few facilities, amenities, or activities to participate in back then.

Third, socially related issues were discussed as obstructions to happiness. Participants generally referred to negative social relations, a decline in social interactions and loneliness as obstructions. For example, Halina heavily relies on being among other people to be happy. She intentionally visits crowded places to see people, as she noticed a decline in her happiness after moving to a more quiet suburban area. Renata refers to her first years in Belgium and marks this as an unhappy period, partly because her entire family still lived in Italy. This, in combination with the language barrier, hampered her social relations. Finally, some participants also pointed to the happiness and health of significant others as a possible obstruction to their happiness. For example, Nico refers to a very unhappy period in his life after learning about his wife’s illness. Isabella elaborates on a similar experience:

“Currently, I am not happy because my son is sick. If my son gets better, I am the happiest woman in the world […] I am not that happy lately. We are a bit … How should I put it? Our life is a bit messed up now.”

     –Isabella (59), Italian migration background

## 4. Discussion

### 4.1. Associations, strategies and obstructions

Given the possible influence of culture and age on individuals’ meaning-making of well-being, this paper explores older migrants’ meaning-making of “happiness”. In line with previous research (Bojanowska & Zalewska, 2016), participants associated “happiness” with health and social relations. In addition, they also associated “happiness” with independence. The latter aligns with Klausen (2020), who points to the increasing importance of independence to people’s well-being as they age. Although there was individual diversity among participants’ meaning-making of “happiness”, participants felt very strongly about the importance of health, social relations and independence. For example, when asked about “happiness”, Elena immediately thinks of “family”. She goes on to explain its importance in her meaning-making of “happiness”. This determination in their meaning-making could perhaps be explained by their more educated needs (Diener et al., 2018).

Participants often referred to negative experiences with their health to emphasize the importance of health to their meaning-making of “happiness”. For example, Liliana discussed the negative effect that her past illness had on her well-being. This highlights the relevance of such health-related reference points in the meaning-making of “happiness” (Noguchi, 2020). In line with this, participants also referred to others’ experiences in discussing the importance of social relations to their “happiness”. For example, some participants referred to their parents’ experiences to position their views on “happiness”. So, in addition to the importance of reference points in general, the results also highlight the important role of others and their experiences as a reference point in making meaning (Manning & Kunkel, 2014) of one’s well-being. Social interactions, in general, were important to participants. They expressed the need for social relations on various levels and intensities. For example, Aicha explained that without that, she would become depressed. This is in line with the literature pointing to the negative effect the loss of social relations can have on life satisfaction (Kodzi et al., 2011). In addition, the need for social relations appears to be cross-cultural (Shin et al., 2018), as participants from various cultures stressed its importance.

Besides highlighting the importance of health and independence to participants’ meaning-making of “happiness”, the intertwinement of both topics became apparent. People are more likely to face health adversities in later life (Jivraj et al., 2014). Some participants experienced this declining health as beyond their control. Parallel, their independence declines through a lack of mobility. Considering the increasing importance of independence as people age (Klausen, 2020), this could impact older migrants’ well-being. For example, the results show that a sense of independence allows participants to participate in personally meaningful activities (i.e., person-activity fit), which seemed important to their “happiness” (Lyubomirsky et al., 2005). The results highlighted that engaging in personally fitting activities is an active strategy to improve well-being. For example, Paola consciously took part in activities she liked to turn a bad day back around. The interviews also highlighted a more passive happiness-pursuing strategy concerning their mindset. For example, participants expressed the importance of being satisfied with what they have. This also included accepting setbacks in life. Whereas Lyubomirsky & Sheldon (2012) discuss the ways to prolong the positive impact of positive life changes (i.e., preventing hedonic adaptation), for example, through continuous appreciation, participants—and older adults in general—were often faced with negative life changes in which they have to appeal to coping mechanisms. For example, Lotka and Jan had to change their mindset and adapt following Lotka’s health adversities. In doing so, they indicated that this did not negatively impact their happiness. Such examples highlight the dynamic, ongoing process of “happiness” meaning-making. Ageing can significantly change participants’ daily lives, which triggers a process of coping and reinterpreting the meaning of “happiness” (Manning & Kunkel, 2014). For example, Fatma indicates that her meaning-making of “happiness” has changed as she ages, stressing the importance of her concern for the well-being of others, an example of changing values as people age (Klausen, 2020).

In discussing obstructions to their “happiness”, participants referred to their migration background. For example, participants referred to the language barrier after migration, which can obstruct older migrants’ well-being (Phlix et al., 2023b). However, most of these migration-related obstructions were situated in the past. Considering the growing importance of the past to older adults’ meaning-making (Halama et al., 2021; Klausen, 2020), these negative migration-related experiences from the past can serve as a reference point to the meaning-making of older migrants’ well-being in the present. Furthermore, although the literature points to the possible positive and negative influence of migrant status on happiness or well-being (Bartram, 2012; Buffel, 2017; Ryff et al., 2003), in Theo’s meaning-making of “happiness”, he considered it a disadvantage, while other participants did not mention a (negative) influence of migration background. Interestingly, although health is a crucial topic in participants’ meaning-making of “happiness”, they did not mention it as an obstruction. So, despite a likely decline in health in later life—which many participants had encountered—it did not seem to impact participants’ well-being negatively (Steptoe et al., 2015). Perhaps this can be explained by their more educated desires (Diener et al., 2018) or passive and active ways to cope with adversities and pursue “happiness”.

So, we identified central themes in older migrants’ meaning-making of well-being. However, the interviews also revealed that participants had very specific, individual and various interpretations of what “happiness” entailed to them. This highlights the importance of considering individuals’ unique positions in and throughout life when assessing their meaning-making of well-being. Closely related to this is the dynamism of well-being throughout life. Some participants referred to unhappy periods in their lives, pointing to the ups and downs in their subjective experience of well-being, as well as the influence of life circumstances on “happiness” (Lyubomirsky et al., 2005; Sheldon & Lyubomirsky, 2019). For example, Massimo and Isabella were experiencing an unhappy period due to their son’s sickness, and Renata refers to her being unhappy right after she migrated to Belgium.

### 4.2. Focusing on ‘age’ and ‘migration background’

Given the focus of this study on older migrants and acknowledging the possible intersecting influences of “age” and “migration”, the data was further analysed with a specific focus on age and migration. In doing so, we reflect on the possible influence of migration background and age on participants’ meaning-making of well-being.

In approaching the results from the perspective of migration, there seem to be some instances in which the migration background of participants influenced their meaning-making of well-being. Some participants referred to their culture of origin in the meaning-making of their well-being. For example, Fatma refers to a Turkish saying to highlight the importance of good relationships with her neighbours. Buffel’s study (Buffel, 2017) also pointed to the importance of neighbours to older Turkish migrants. Another example concerns religion, which seemed important in some participants’ meaning-making of well-being. Especially Muslim participants—whose religion differs from the dominant religion in Belgium—were referred to religion in the meaning-making of their “happiness”. This aligns with Kodzi et al. (2011) who state that religion is a lens through which people approach their lifestyle. For example, values and norms deemed important in the Islamic religion (i.e., not being selfish, helping others) served as an important reference point for Marouan’s meaning-making of “happiness”. This explicit link between the meaning-making of “happiness” meaning-making and religion was made mainly by Muslim participants, hinting at the non-universal effect of religion on people’s well-being, highlighted by Diener et al. (2018). Here, a religion-environment fit (i.e., religious people in a religious environment) is of the essence, especially considering its impact on well-being (Diener et al., 2011). As most participants who have an Italian or Polish migration background are Catholic, to some extent, they were able to experience a religion-environment fit in the historically Catholic-oriented Belgium.

Overall, in asking older migrants about their associations and meanings of “happiness” and inductively analysing their answers, aside from some examples concerning culture and religion, the role of migration background did not emerge as a prominent theme. Nevertheless, we want to stress that migration background is inherently part of participants’ identities. In line with Torres (2007), we argue that culture and migration background can serve as a reference frame for older migrants to give meaning to well-being. However, following this argument, one should be mindful of not only the culture of origin as a reference frame. Considering most participants have lived in Belgium for over 40 years, the Belgian culture also becomes part of older migrants’ reference frame to give meaning to well-being. Both the argument of the (lack of) religion-environment fit and the role of migration background as a reference frame highlight the importance of adopting a time-sensitive perspective in analysing this matter. This is again highlighted in approaching the results from the perspective of ageing. For example, as participants age, health and independence have become more important in their associations of “happiness”. So, age seems to influence participants’ meaning-making of well-being in later life, which aligns with previous research (Laaksonen, 2018; Oishi & Gilbert, 2016). To understand this trend, participants’ life course experiences are relevant. Considering participants’ life course, including the many years spent in Belgium, could explain the limited impact of migration background on participants’ meaning-making of well-being. For example, this could explain the more present role of migration background when participants were discussing “happiness” obstructions. In doing so, they referred to their past experiences in which there was a large discrepancy between their reference frame (i.e., culture, religion) and that of the receiving society.

However, we argue that in exploring life course experiences, instead of isolating migration background as a sole influence on older migrants’ conceptions of “happiness”, one should be mindful of various characteristics across the entire life course. For example, migrants’ immersion in the culture of the country of settlement could create a dual reference frame in which the meaning-making of well-being occurs. This could explain Renata’s wish to live in Belgium and Italy. Another example of the life course concerns, as the results show, the increasing importance of health to participants’ happiness as they age and experience declining health. This could be explained by the associated decline in well-being as health is compromised in later life (Jivraj et al., 2014). The influence of ageing also seeps through in the importance of social relations to participants’ meaning-making. Steptoe et al. (2015) point out that social relations change as people age. The possibility of a shrinking social network rises with age, negatively affecting older adults’ life satisfaction (Kodzi et al., 2011). Consequently, this could explain participants’ strong emphasis on the importance of having qualitative social relations. This is again related to health, as social relations can become increasingly important in providing support. Moreover, for some participants, this also intersects with their migration background. For example, as a consequence of migrating, Aicha has no family in Belgium. So, the results indicate the complex interrelatedness of various variables in participants’ meaning-making of “happiness”. For example, the aforementioned “happiness” associations (i.e., health, social relations and independence) seem intertwined and both age and migration background—albeit to different degrees—affect the meaning-making reference frame of participants.

## 5. Conclusion

Through interviews with older adults with a migration background, this study has identified some central associations in their meaning-making of subjective well-being (i.e., health, social relations and independence), which seem closely intertwined. In addition, the interviews also highlighted participants’ views on “happiness”-pursuing strategies (both active and passive) and barriers to obtaining “happiness” (either migration-, agency- or socially related).

The results were approached from a perspective of ageing and migration, unveiling the different impact of both variables on participants’ meaning-making of “happiness”. Aside from religious influences—prevalent primarily in Muslim participants’ accounts—and past experiences related to migration background serving as a reference frame to the meaning-making of well-being, the influence of migration background remained rather limited. On the contrary, the influence of age was quite prominent in participants’ meaning-making of “happiness”. For example, participants’ well-being associations were imbued with age-related meanings that also increased in importance as participants aged. This difference between the influence of age and migration could be explained by considering participants’ life course experiences. As the importance of the life course came up inductively during the analysis of the results, we suggest that a more thorough analysis adopting a life course perspective (Elder, 1994, 1998) while also acknowledging the intersectional and unique position of individuals could be promising in understanding individual variations in the meaning-making of well-being.

As this study mainly focused on age and migration, exploring other (intersecting) life events that affect people’s meaning-making of well-being could be valuable. Moreover, this study has taken place in a specific local and national context relevant to the migration context, as well as the meaning-making, terminology and conceptualization of subjective well-being (or: “happiness”). Therefore, the results of this study should be understood from this limited context. This study’s results are also limited to a rather small but especially diverse sample of participants. Therefore, for future research, it would be interesting to explore larger samples of participants from various ethnic backgrounds.

## Notes

1. A “general term referring to the various types of subjective evaluations of one’s life, including both cognitive evaluations and affective feelings” (Diener et al., 2018, p. 3).

2. People who migrated at a young age, as a child (Bilecen et al., 2022).

3. Older migrants living in residential care facilities were excluded as the overarching research project focuses on “ageing in place”.

4. Aicha’s exact age is unknown because of unclear birth registrations in Morocco.

5. Isabella meets all search criteria but one (i.e., aged 60 and older). However she was included as she sat in on the interview with her husband Massimo and provided interesting insights.

6. Inge has migrated from the Netherlands to Belgium (1^st^ generation) and is a second-generation Indonesian migrant following her mother’s migration to the Netherlands.
